# Precision Radiology: Predicting longevity using feature engineering and deep learning methods in a radiomics framework

**DOI:** 10.1038/s41598-017-01931-w

**Published:** 2017-05-10

**Authors:** Luke Oakden-Rayner, Gustavo Carneiro, Taryn Bessen, Jacinto C. Nascimento, Andrew P. Bradley, Lyle J. Palmer

**Affiliations:** 1 Department of Radiology, Royal Adelaide Hospital, North Terrace, Adelaide, SA 5000 Australia; 2 School of Public Health, The University of Adelaide, North Terrace, Adelaide, SA 5000 Australia; 3 School of Computer Science, The University of Adelaide, North Terrace, Adelaide, SA 5000 Australia; 4 Instituto Superior Técnico, Lisbon, Portugal; 5 School of Information Technology and Electrical Engineering, The University of Queensland, Building 78, St Lucia QLD 4067, Queensland, Australia

## Abstract

Precision medicine approaches rely on obtaining precise knowledge of the true state of health of an individual patient, which results from a combination of their genetic risks and environmental exposures. This approach is currently limited by the lack of effective and efficient non-invasive medical tests to define the full range of phenotypic variation associated with individual health. Such knowledge is critical for improved early intervention, for better treatment decisions, and for ameliorating the steadily worsening epidemic of chronic disease. We present proof-of-concept experiments to demonstrate how routinely acquired cross-sectional CT imaging may be used to predict patient longevity as a proxy for overall individual health and disease status using computer image analysis techniques. Despite the limitations of a modest dataset and the use of off-the-shelf machine learning methods, our results are comparable to previous ‘manual’ clinical methods for longevity prediction. This work demonstrates that radiomics techniques can be used to extract biomarkers relevant to one of the most widely used outcomes in epidemiological and clinical research – mortality, and that deep learning with convolutional neural networks can be usefully applied to radiomics research. Computer image analysis applied to routinely collected medical images offers substantial potential to enhance precision medicine initiatives.

## Introduction

## Measuring phenotypic variation in precision medicine

Precision medicine has become a key focus of modern bioscience and medicine, and involves “prevention and treatment strategies that take individual variability into account”, through the use of “large-scale biologic databases … powerful methods for characterizing patients … and computational tools for analysing large sets of data”^1^. The variation within individuals that enables the identification of patient subgroups for precision medicine strategies is termed the “phenotype”. The observable phenotype reflects both genomic variation and the accumulated lifestyle and environmental exposures that impact biological function - the exposome^2^.

Precision medicine relies upon the availability of useful biomarkers, defined as “a characteristic that is objectively measured and evaluated as an indicator of normal biological processes, pathogenic processes, or pharmacological responses to a therapeutic intervention”^3^. A ‘good’ biomarker has the following characteristics: it is sensitive, specific, predictive, robust, bridges clinical and preclinical health states, and is non-invasive^4^.

Genomics can produce good biomarkers useful for precision medicine^5^. There has been significant success in exploring human genetic variation in the field of genomics, where data-driven methods have highlighted the role of human genetic variation in disease diagnosis, prognosis, and treatment response^6^. However, for the chronic and age-related diseases which account for the majority of morbidity and mortality in developed nations^7^ and worldwide^8^, the majority (70–90%) of observable phenotypic variation is related to non-genetic determinants^9^. It has become clear that physical biomarkers more proximal to the outcome of ultimate interest (usually clinical morbidity or mortality) are needed in addition to genomic markers. To date, such biomarkers have proven difficult to capture and analyse for precision medicine purposes.

Environmental exposures are difficult to quantify because they occur outside of the medical context, accumulating and changing throughout life^10^. Environmental exposures such as diet and physical activity are also notoriously difficult to measure objectively or retrospectively^11, 12^. Rather than trying to measure environmental exposures directly, “it makes sense to employ a top-down approach based upon biomonitoring (e.g. blood sampling) rather than a bottom-up approach that samples air, water, food, and so on”^10^. Current clinical and research methods of biomonitoring explore the phenotype (the combination of genetics and exposures) by assessing the patient’s clinical history, examination findings, and test results^13^. Aggregated, large-scale clinical data can be analysed to identify factors (biomarkers) that correspond to important variations in health in a similar way to that which already occurs in genomics^14^. Current national and international efforts seek to expand these databases to include other “-omic” information, such as proteomic and microbiomic data^1, 15–21^ (the high-throughput analysis of endogenous body proteins and commensal organisms respectively).

An important potential high dimensional source for phenotypic data and biomarkers are data collected for routine clinical purposes^22^. However, the types of the data commonly used for this application may not currently be well suited for precision medicine for several reasons. Unlike genomics, where the data is replicable, high quality, and objective, clinical information is sometimes incomplete, of variable accuracy, and highly complex^14^. Clinical data also rarely captures asymptomatic pathological variation; chronic diseases often develop slowly and silently over decades without any direct clinical measurement. That is, important biomarker variation is usually both unmeasured and latent before frank disease in diagnosed. Much of the important variation in health measures that precision medicine would wish to capture, particularly for prognostic purposes, is thus *pre-clinical*. Furthermore, collecting the extensive medical histories, examination findings, and test results needed for accurate clinical phenotyping can be both costly and time-consuming if such collection and aggregation in well-curated databases is outside of routine practice^23, 24^. Finally, the current use and interpretation of clinical tests may be non-optimal for describing human health because of our incomplete understanding of disease^25^, as we cannot design tests for processes we do not understand.

To expand on this last point, in mathematics and computer science a method is said to be *optimal* if it performs better than other methods at the desired task according to some criterion^26^. In mathematics, the solution space for an optimisation problem can usually be explored completely. For example, in a problem where the solution will be any integer number we could theoretically test every candidate solution, although this would be computationally inefficient. In medical testing we cannot explore every solution; we are limited to a set of biomarkers we have already discovered and validated. This set of biomarkers is limited by the technical capabilities and knowledge of the day. As an example, a biomarker of myocardial infarction (MI) known as CK-MB was used for decades until a number of scientific and technical advances led to the development of the troponin assays^27^. Troponins were found to be superior for the assessment of MI, and have superseded the use of CK-MB testing in almost all settings^27^.

The suboptimality caused by incomplete biomarker knowledge and testing capacity is less problematic for high-throughput ‘-omic’ methods because these approaches are generally “hypothesis free” and do not rely on previously discovered biomarkers. These methods attempt to comprehensively explore an entire domain of biomarkers. Genomics explores the entire genome in a hypothesis free way, nucleotide by nucleotide. Similarly, proteomics explores the entire set of proteins that are created by the body. The best performing biomarker can be seen to be the optimal choice for that task within each domain. To return to the earlier example, comprehensive testing of endogenous proteins with proteomics techniques in the setting of MI could have demonstrated that troponins outperform CK-MB. Without proteomics this discovery required more than twenty years of research^27^. It is also plausible that further unknown latent protein biomarkers of MI may in turn outperform troponins: a proteomic analysis could also discover these new markers. Despite this advantage, phenotyping with ‘omic’ methods has been largely unsuccessful to date in producing new diagnostic, prognostic, or treatment response biomarkers and risk models^28^. These technologies appear unlikely to offer comprehensive diagnostic or prognostic biomarker discovery solutions for most chronic diseases because of their limited domains; proteomics will only be relevant if variation in health alters serum protein levels, microbiomics when there is an altered balance of commensal flora, and so on.

In summary, the promise of precision medicine relies upon the discovery and use of good biomarkers for health and disease. Currently available biomarkers have limitations and can be problematic for use in precision medicine applications. In particular, genomics and other ‘omic’ domains have not yet fully delivered on promises *apropos* diagnostic and prognostic markers^29^, and currently utilised clinical data is limited by quality, optimality, and application issues. In this context, it is critical to develop new methods to assess phenotype variation more objectively, accurately and comprehensively for precision medicine tasks. Since any observable physical phenotypic variation in human health must correlate to tissue changes at the cellular or subcellular level, an ideal precision medicine test would assess the key areas of the body or the whole body with microscopic resolution, quantify the tissue changes of clinical and pre-clinical disease, and identify optimal biomarkers of health variation. These biomarkers should be easily and cheaply obtained (ideally collected for routine clinical purposes), objective and reproducible. Such biomarkers will also be increasingly important for so-called “phenome-wide association studies”^30^.

With these goals in mind, we propose that images derived from routine radiological testing have been largely ignored in the context of precision medicine, and motivate the use of powerful new machine learning techniques applied to radiological images as the basis for novel and useful biomarker discovery.

## The role of medical imaging in precision medicine

Medical images are routinely collected and contain dense, objective information. Medical image analysis is therefore highly attractive for precision medicine phenotyping. Cross-sectional studies can comprehensively assess whole regions of the body during a single examination, a variety of human and machine detectable changes have been shown to quantify clinical and preclinical disease states^31–33^, and high-throughput image analysis techniques may be able to identify biomarkers which are closer to optimal for a given task. Recent advances in the field of medical image analysis have shown that machine-detectable image features can approximate the descriptive power of biopsy, microscopy and even DNA analysis for a number of pathologies^34–36^.

‘Radiomics’ as a field can be broadly defined as the use of high-throughput computational techniques to analyse the high-dimensional data of medical images^34, 37, 38^. There are two image analysis methods currently considered state of the art in their respective fields that are applicable in the radiomics domain: “traditional” image analysis with human-defined image features, and deep learning with feature learning. We note that the human-defined feature method is currently synonymous with the term “radiomics” in some of the relevant literature, however we suggest that a broader definition of the field is more useful than a narrow focus on a single method.

Traditional image analysis methods with human-defined features have been utilised in the radiology and informatics communities and have shown significant promise in finding useful biomarkers of health, particularly in cancer subclassification^34–36^. However, these techniques have never been applied more broadly to the phenotyping of overall health and latent disease. These methods attempt to identify and analyse a large number of candidate biomarkers at once. Candidate biomarkers are termed “image features”; mathematical descriptions of the visual properties of an image informed by decades of research in computer vision^39^. These human designed or “engineered” image features typically describe the low level visual information present in an image, such as the intensity/brightness and the texture of image regions. These methods are limited because they require expert input (e.g., a radiologist) to define *high-level* features. High level features are complex patterns in the images which are semantically relevant to the task. In the case of medical images, such features are those a human expert recognises in the practice of diagnostic radiology, for example the presence or absence of a lung cancer. In general, these abstract concepts are almost impossible for a human expert to define mathematically, and so this is an unsolved problem. However, in some specific applications high-level features are amenable to mathematical definition, or can be constructed by combining multiple low level features. These form the image biomarkers we currently use today (e.g., counting the number of high density pixels in the wall of an artery to quantify the atherosclerotic plaque burden). Even when this is possible, the process is very time-consuming and limited by the knowledge of the expert. A further weakness of using human-defined features for to image analysis is the need to perform feature design, extraction, selection, and combination as discrete steps, where each step introduces new biases and complicates the analysis.

The current trajectory of methods in traditional, human engineered image analysis is broadly analogous to that of the history of human gene discovery. Quantitative scoring systems (such as coronary artery calcium scoring) are similar to gene discovery attempts using candidate genes defined on the basis of prior biological knowledge. The use of human-designed “agnostic” feature sets (e.g., generic intensity, shape and texture features) are similar to the early investigation of single nucleotide pleomorphisms (SNPs), where the sets of SNPs genotyped provided only partial coverage of the genome, i.e., were only a small subset of all possible SNPs and an even smaller subset of all possible genetic variants. With few exceptions, such early efforts in genetics did not discover robust, replicable genetic variants associated with chronic disease^40^. Rather, it has been the advent of whole genome association scans and next generation sequencing used in a hypothesis free paradigm that has enabled the discovery of thousands of novel genetic variants robustly associated with chronic diseases^41^. Analogous to this, novel biomarker discovery in radiomics now requires robust, hypothesis free approaches that analyze all of the available variation in an image. Deep learning appears to be an ideal tool to accomplish this goal.

Deep learning has developed from the computer science community and has rapidly overtaken more traditional methods in many computer vision tasks, such as image recognition and segmentation^42^. In fact, deep learning systems have approached or even surpassed human level capabilities for complex “real-world” tasks such as image recognition, speech recognition, natural language processing, complex game playing and more^42^. Deep learning *automatically* discovers visual features that are suited to a specific task through a process of optimisation, including both low-level and high-level features^42^. Deep learning can therefore offer an exciting solution to incomplete biomedical knowledge: these methods can discover new biomarkers without any human input, conceivably generating truly unexpected discoveries. Deep learning image features can be later visualised, providing human-understandable explanations for the deep learning models^43^. Currently, a weakness of deep learning methods is the limited validation of these methods in medical imaging, although recent exciting results have demonstrated similar performance to human experts in the assessment of diabetic retinopathy and dermatological lesions^44, 45^. In particular, the application of these methods to assess the tissue changes of chronic disease has not been explored.

A comparison between clinical data, genomic data, and image analysis methods for biomarker discovery methods is presented in Table 1.

**Table 1:** Comparison of features of clinical data, genomic data, traditional feature engineering, and deep learning image analysis methods as potential phenotyping solutions for precision medicine.

Desirable feature	Phenotyping/biomarker discovery method			
	*Clinical data*	*Genomic data*	*Traditional image analysis*	*Deep learning image analysis*
Broad phenotypic coverage	No	Assesses lifelong risk only	Yes	Yes
Accessible data	Messy and complicated^14^	In development (e.g., PMI cohort)	Large centralised image archives	Large centralised image archives
Sensitive to preclinical tissue changes	No	Assesses lifelong risk only	Yes	Not yet explored
Optimal biomarker discovery	No	Optimal genomic features	Optimal low level image features	Optimal low level *and* high level image features
Useful in chronic disease	Yes	Limited use (10–30% of risk)^9^	Not yet explored	Not yet explored

## Investigating image analysis to assess health phenotypes

We present a preliminary ‘proof of principle’ study to assess the utility of medical imaging for health phenotyping. We investigate the analysis of routinely conducted CT chest imaging in adult patients (age >60) to predict longevity. We chose longevity as our primary outcome as mortality represents an easily obtained and well-defined outcome (in this case, 5 year mortality rate), and strongly correlates to the underlying presence of chronic disease^46^. The strong relationship between age, morbidity, and mortality motivates the selection of near-term mortality as a surrogate biomarker for overall health status. CT chest scans were chosen for several reasons. CT scans are the most highly utilised cross-sectional medical imaging studies with an estimated 2.1 million CT scans performed in Australia in 2009^47^ and 69 million CT scans performed in the USA in 2007^48^. CT scans are also the least variable cross-sectional imaging modality due to the standardisation of pixel values and the defined relationship between the pixel values and the physical density of the tissue^38^. Finally the thorax contains tissues directly involved in much of the morbidity and mortality in older adults (i.e., the lungs, heart, great vessels, and other organs). Many of the existing quantitative imaging biomarker methods related to chronic disease and mortality utilise CT chest imaging^31, 32^.

Mortality prediction has been attempted previously using clinical data, including survey results and laboratory tests^24, 49^. While promising accuracy has been achieved with these methods, the tools have not seen widespread adoption^24^. There has been minimal work to integrate medical image data into systems to predict mortality.

We investigate both traditional image analysis with human-defined features and deep learning techniques (“convolutional neural networks”) for this task, as these techniques have different strengths and weaknesses. In particular the traditional image analysis methods have been previously validated as a method of describing subtle tissue changes, and deep learning offers the automatic and hypothesis free learning of complex and high-level image features.

We present the novel application of these methods to the prediction of longevity in older adult patients as evidence to motivate the application of radiomics techniques to medical images in order to produce biomarkers for precision medicine applications.

## Results

### Exploratory analysis of human-defined feature methods

**Figure 1:**
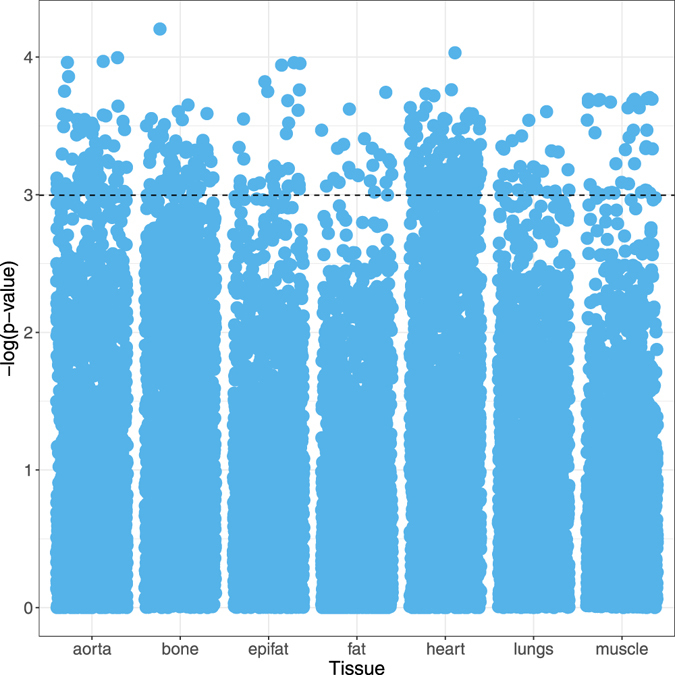
Manhattan plot showing the distribution of covariates in each tissue segment. The dotted line identifies the threshold of significance (the −log_10_ of the p-value is plotted on the y-axis, covariates above this line have p-values < 0.05).

A total of 15,957 CT image features from seven tissue segments were defined by an expert radiologist and extracted from each study. Univariate conditional logistic regression revealed 417 out of the 15,957 image features (2.6%) were associated (P < 0.05) with 5-year mortality. The distribution of association test results within each tissue segment is shown in Fig. 1.

The general under-representation of image features at the conventional threshold of significance is likely due to the co-dependence between the features extracted and is an inherent limitation of the human-defined feature methodology. That is, the number of independent features is significantly less than 15,957. We do identify proportionally more significant features in the tissues (segments) that are most often associated with mortality risk in the literature – the heart (9.8% of heart features), the aorta (8.5% of aorta features) and the bone (10.6% of bone features). The bone tissue segment also demonstrated a high proportion of features informed by the previous biomarker literature (i.e., evidence-based features – see methods) that reached the threshold of significance (41% of total evidence-based bone features). The distribution of evidence-based and task-agnostic features is shown in Supplemental Figure 1.

### Mortality risk phenotypes using human-defined features

**Figure 2:**
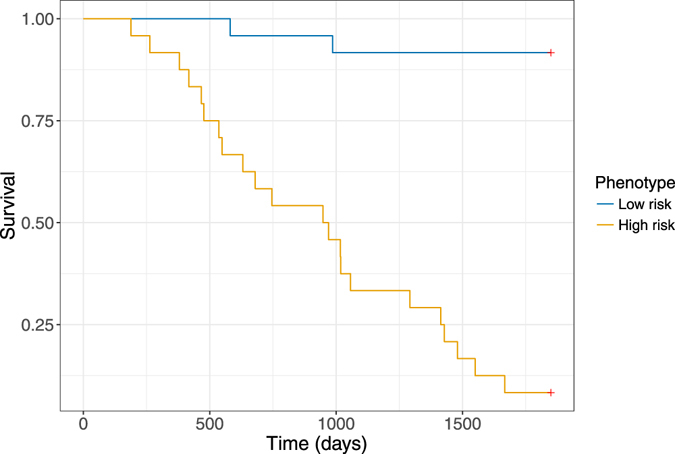
Kaplan-Meier curves demonstrating 5-year survival for the high and low risk phenotypes developed using human engineered image features extracted from CT chest scans. There were two mortalities for the twenty five patients identified to be low risk, and twenty mortalities amongst the twenty three patients identified as high risk. The difference between curves is significant (p < 0.000005).

We created a predictive model using multivariable survival analysis (Cox regression). The model was informed by the top 5 covariates selected by minimum redundancy-maximum relevance feature selection^50^. These covariates were standardised, and the resulting risk score was dichotomised at the mean to create high-risk and low-risk phenotypes. Table 2 shows the 5-year mortality rate for high and low risk phenotypes, and the related Kaplan-Meier curves are presented in Fig. 2. The difference between the survival curves for of the high and low risk phenotypes is highly significant (p < 0.00005). The distribution of the raw mortality phenotype scores among cases and controls are presented in a box and whisker plot in Supplemental Figure 2.

**Table 2:** Mortality rate for the dichotomised risk phenotype developed using human engineered image features extracted from CT chest scans. There was no censoring.

Risk phenotype	n	5-year mortality rate
Low risk	25	8%
High risk	23	87%

### Machine learning 5-year mortality predictions

**Figure 3:**
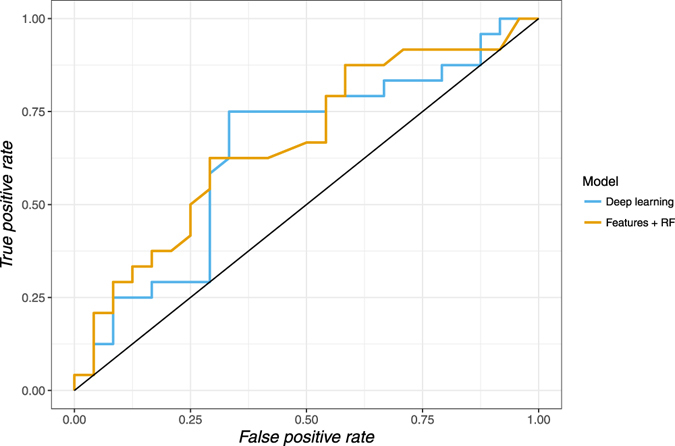
Pooled ROC curves of the 6-fold cross validation experiment to predict 5 year mortality and survival outcomes from CT chest imaging, comparing the deep learning model and the human feature engineering/random forest model.

Five-year mortality prediction was performed with deep learning, as well as a range of classifiers trained on the human-defined image features. Classification models tested included random forests, support vector machines and boosted tree algorithms. The random forest model performed the best of the human-defined feature classifiers. This was an expected result, as random forests are known to perform well in smaller data settings as they are fairly robust to noise^51^. We show the pooled ROC curves (combining 6 cross validations) for the testing sets of the deep learning and the best traditional models in Fig. 3. In Table 3 we present the mean and the standard deviation of the AUC and accuracy results for the deep learning and the best traditional models. Comparison is also made with selected published clinical scores predicting 5 year mortality.

**Table 3:** Mean and standard deviation of the AUC and accuracy results of the 6-fold cross-validation experiment using the deep learning and human engineered feature models, compared to previously described clinical prognostic scores.

Study	Analytic model	AUC (mean ± std dev)	Accuracy (mean ± std dev)
Current study	Deep Learning	0.677 ± 0.214	0.687 ± 0.153
Current study	Human engineered features	0.646 ± 0.255	0.646 ± 0.123
Schonberg *et al*.^24^	Clinical features	0.75*	—
Ganna *et al*.^49^	Clinical features	0.79–0.80*	—

^*^Clinical risk score results were reported as c-index rather than AUC, although in this context these terms are interchangeable.

Using a t-test for paired samples there was no significant difference between the accuracy (p = 0.61) and AUC (p = 0.62) of the deep learning and best human-defined feature models. Both the deep learning model and the best human-defined feature model demonstrated a significant improvement in accuracy compared to the null (i.e., accuracy = 0.5) (p = 0.03 using a one sample t-test for both methods) although neither method demonstrated formal significance for the AUC measurement (p = 0.09 and p = 0.22 respectively).

**Figure 4:**
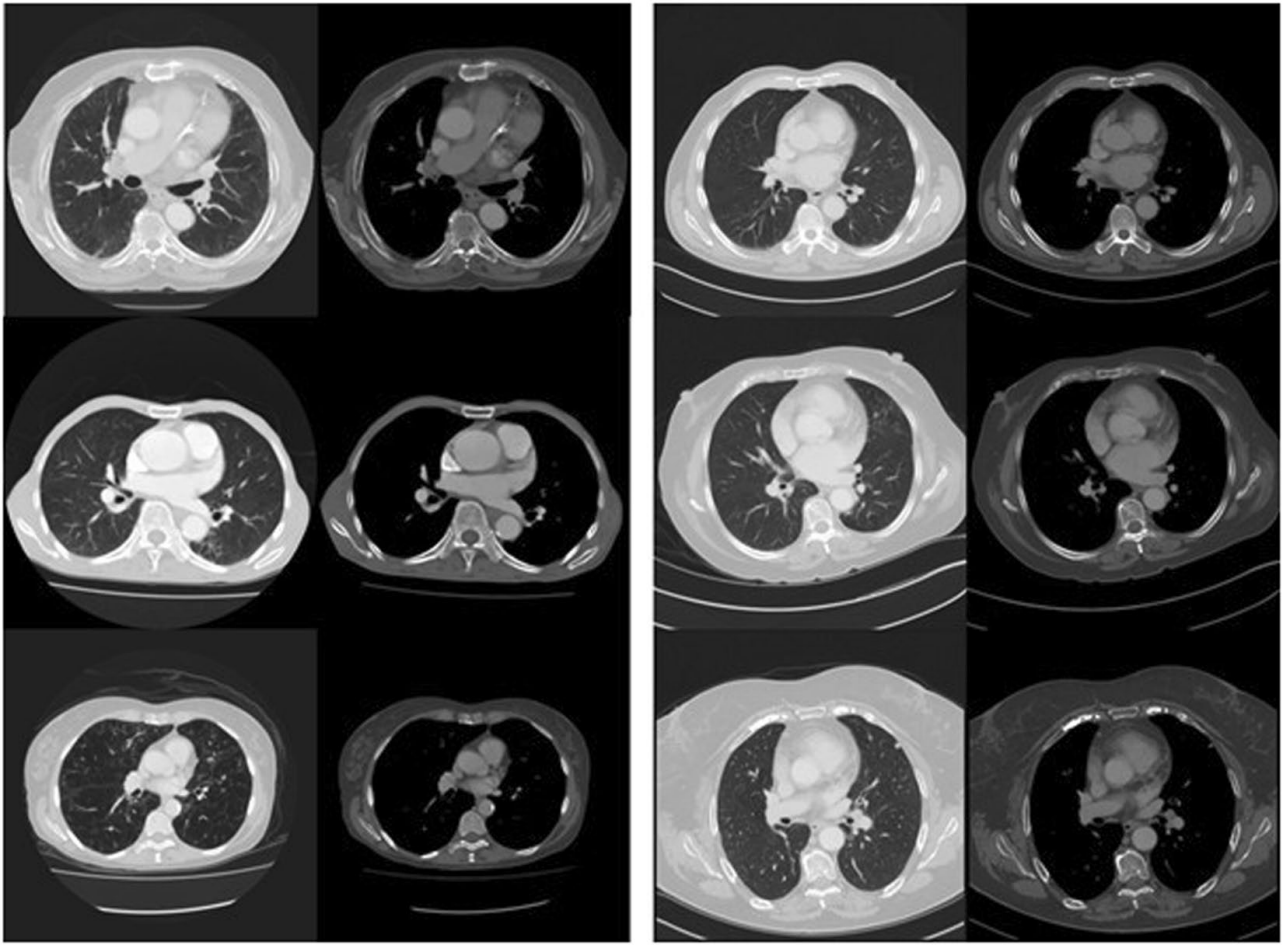
Images at the level of the proximal left anterior descending coronary artery, with the most strongly predicted mortality and survival cases selected by averaging the predictions from the deep learning and engineered feature models. The mortality cases (left side) demonstrate prominent visual changes of emphysema, cardiomegaly, vascular disease and osteopaenia. The survival cases (right side) appear visually less diseased and frail.

Finally, qualitative visual assessment of the CT chest images was performed. The highest certainty average predictions of the deep learning and engineered feature models for survival and mortality outcomes were examined by a consultant radiologist for visual differences. This revealed many of the expected associations; patients predicted to survive longer than five years appeared visually healthier than those predicted to die within 5 years. In Fig. 4, we show representative images from the three chest CT scans with the highest certainty predictions for mortality and survival.

## Discussion

The overall goal of precision medicine is to inform useful predictive models of health and disease. We have presented a novel application of medical image analysis as a proof of concept and to motivate the use of routinely collected, high resolution radiologic images as sources of high quality data for precision medicine. We propose that the comprehensive assessment of tissues with cross-sectional imaging may better reflect the underlying microscopic changes occurring in the body than current clinical and laboratory methods, and that the contemporary nature of patient imaging can explore the combination of genetic and environmental risks better than genome testing alone. Biomarkers derived from images such as those described in this paper have the additional advantages of directly informing research with regards to important variation in critical organs and tissue, and of being more proximal to the ultimate outcome of interest - disease or health - than the features measured by some other technologies such as genomics (i.e., variation in DNA sequence).

We have shown the first proof of concept experiments for a system that is capable of predicting 5-year mortality in older (age >60 years) individuals who have undergone chest CT imaging. The results are promising, with prediction accuracy from routinely obtained chest CT images alone similar in our study for both the current state of the art ‘human engineered’ approach and for deep learning. The predictive accuracy we obtained was also broadly similar to previously published human engineered clinical risk scores^24, 49^. However, comparing our study design to previously published studies is challenging for several reasons. Our experiments are retrospective, with evenly distributed mortality and survival cases, and controlled for highly predictive covariates such as age and gender. The majority of published studies using clinical biomarkers have been prospective, and demonstrated mortality rates in their study populations between 5 and 30%. Furthermore, the choice of comparison studies was complicated by the lack of standardised experimental designs. Previous studies enrolled patients from different patient groups (e.g., screening adult population vs. hospital admissions vs. nursing home residents), and reported different follow-up times (between 1 year and 6 years). Each of these studies incorporated covariates such as age and sex in their models, which were shown to be highly predictive. These limitations hamper the comparison between prognostic indices in different studies^24^. In Table 3 we compare our model against the two studies that have the most similar follow-up periods, similar patient cohorts and the most predictive models, which we feel is the fairest and most conservative comparison we could present. As can be seen, our models provide broadly similar predictive accuracy.

Our classification results (c-index 0.68) using only a CT image also compare favourably to clinical prognostic scores currently in widespread use, such as the Framingham risk model for lipid therapy decision support (c-index 0.63–0.83)^52^, the CHADS2 score for the use of warfarin therapy (c-index 0.68–0.72)^53^, and the TIMI score used to assist in invasive treatment choices in unstable angina (c-index 0.65)^54^. Note that in comparing these results, the terms AUC and c-index are interchangeable^55^.

Our proof-of-concept study is limited by the modest size of our dataset, particularly as the techniques employed are designed to be optimal with large volumes of data^42, 56^. This concern is at least somewhat mitigated by the convergence of our findings using both human-defined feature analysis and deep learning methods with automated feature learning. Furthermore, we note evidence of ‘face validity’ on qualitative image review – the strongly predicted mortality and survival cases demonstrate the expected patterns of disease when analysed by an expert radiologist. The modest size of our data set is currently unavoidable in this area of research. Segmentation of multiple anatomic structures requires high level medical expertise and takes a prohibitive amount of time, so a larger dataset was unfeasible in a preliminary study. Despite these limitations, to the best of our knowledge, our training dataset is the largest of its kind in the world.

The current limitations on sample size are expected to be alleviated in the near term. Automated segmentation methods are improving rapidly, particularly with the application of deep learning methods^57^. Furthermore our models are fairly robust to minor segmentation errors; the current state-of-the-art segmentation methods produce errors on the order of tens of pixels, which is less significant at the scale of whole organs compared to the more often attempted tumour and organ sub-region segmentation tasks. We are currently working with our dataset to create automated deep learning segmentation models, with significant success (preliminary results demonstrated in Supplemental Figure 3). We believe these early results show that the segmentation challenge is tractable, and we envision a time in the near future where radiomic analysis of whole tissues will be near instantaneous. When this is achieved, datasets can be as large as the available medical imaging archives. We are now extending this work to a much larger dataset (n > 12,000).

While the segmentations are necessary to apply the human designed feature techniques, the benefit is less clear for the deep learning approach. We provided the segmentation information to the deep learning system with the assumption that anatomical priors would be useful when automatically learning features related to mortality, particularly when dealing with a small dataset. This assumption will be explored in future work; it may be that segmentation masks are not required for well-trained deep learning models.

A further limitation of this work is the lack of statistical adjustment for diagnosed disease. We could not explore this due to our small sample size. This does not weaken our findings as our goal was to explore the use of radiomics methods to assess health, which is a combination of both diagnosed and undiagnosed pathology. While we cannot discriminate between the relative contributions of diagnosed and undiagnosed disease in this study, the ability to identify “healthy” and “unhealthy” patient subgroups remains an exciting and novel contribution.

We have presented multiple methods in this paper as there is no pre-existing gold standard method; the task of general health assessment and outcome prediction using medical imaging is currently under-explored. We have implemented several state-of-the-art radiomics methods; both human-designed feature analysis and deep learning approaches. Human-designed feature methods have been validated for a subset of medical image analysis tasks in the radiology literature in recent years, and our implementation and adaptation of these methods will provide a useful baseline measure for future comparison. Deep learning methods hold much promise in medical image analysis, and as shown in our results even a naïve application of these methods to our complex and multifactorial predictive task appears to perform at least on par with the human-designed feature approach. This is all the more impressive considering the input data for the deep learning models was heavily down-sampled due to computational constraints. We believe that this finding is important for researchers and practitioners in the field of medical imaging, as it suggests that the human-designed imaging features which have been informed by decades of previous medical and computer science research may be no better than features learnt automatically from the data in an hypothesis free approach.

The possible synergy between these techniques and other methods is also attractive. We excluded highly predictive clinical covariates from our analysis to investigate the specific capabilities of medical imaging analysis, but a combination of our techniques with several readily available clinical covariates such as age, gender, and ethnicity is likely to yield significantly improved predictive capability. The published clinical mortality scores derived from radiologic image analysis not only suggest that these covariates significantly improve the performance of predictive models, but also show they are among the most predictive clinical features^24, 49^. The possibility of combining our techniques with genomics analysis is also likely to yield further improvements, representing a more complete and quantitative assessment of phenotype (i.e., the observable results of genome and exposome).

Finally, the widespread use of high resolution medical imaging of the internal body in routine clinical practice suggests that our methods, once successfully tested in large scale datasets, could be translated with relative ease to clinical use as the only required inputs – the medical images - are already readily available and highly utilised. This is particularly noteworthy when compared to previous clinical predictive scores that require additional complex data collection, such as targeted clinical histories, clinical examinations and multiple laboratory tests^24, 49^. With radiomics models, allowing for the potential need for automated segmentation approaches, accurate predictions could be obtained with little additional marginal costs in finance or time. We also note that these methods are likely to be applicable to the prediction of other important medical outcomes (e.g., the prediction of treatment complications), and to other modalities of high resolution routine imaging (e.g., MRI).

## Conclusion

Precision medicine aims to tailor treatment to specific patient sub-groups or individuals but has been held back by the lack of simple, accurate, and replicable quantitative methods to discover and utilize novel biomarkers needed to assess the full range of phenotypic variation. We present a conceptual framework for the use of radiomics techniques with clinically conducted cross-sectional scans to identify tissue-wide pathological changes (imaging biomarkers) that quantify the latent health of the patient. We also demonstrate that modern deep learning techniques can be used within a radiomics framework, and perform at least as well as more traditional, hand-engineered approaches. Our preliminary proof-of-principle findings are promising and offer a path towards an effective and efficient testing methodology that can accurately measure the widespread tissue changes predictive of chronic diseases. This testing could quantify preclinical disease, inform treatment choices, and guide research cohort selection.

## Methods

### Ethical statement

Use of medical imaging and mortality data and the experimental protocol were approved by the Royal Adelaide Hospital Human Research Ethics Committee. All experiments were carried out in accordance with relevant guidelines and regulations.

The study utilised only pre-existing medical data, therefore patient consent was not required by the ethics committee.

### Study design and participants

To evaluate the base predictive value of medical imaging using traditional human feature engineering image analysis and deep learning techniques we performed a retrospective case-control study, with matching used to control for non-imaging clinical and demographic variables that were expected to be highly predictive of five-year mortality.

Participants were selected from the Royal Adelaide Hospital (RAH) radiology department archive. All demographic information used in the study was obtained via the DICOM metadata fields of the related imaging studies. Mortality data was obtained via the RAH case-mix department, which receives mortality information for RAH patients from the South Australian Registrar of Births, Deaths and Marriages.

We enrolled 37 sequential decedents with mortality in 2014, who had undergone CT chest scans in the 5 years preceding death and were older than 60 years at the time of imaging. Participants were excluded based on the following criteria: acute visible disease identified on CT chest by an expert radiologist, metallic artefact on CT chest, and active cancer diagnosis (which would strongly bias survival time). A total of 13 cases were excluded, for a total of 24 cases included in the cohort.

24 controls were matched on age, gender, and source of imaging referral (emergency, inpatient or outpatient departments), for a total of 48 image studies included (24 who died within five years of imaging, and 24 who survived beyond five years). The same exclusion criteria were applied. This pair matched study design was utilised to explore the predictive ability of image analysis techniques in this setting without major confounding.

### Image data

Post contrast CT chest scans were obtained using three types of CT scanners (GE Picker PQ 6000, Siemens AS plus, and Toshiba Aquilion 16) using standard CT protocols at the RAH. CT chest images were obtained in the late arterial phase, following a roughly 30 second delay after the administration of intravenous contrast.

Thick section (5 mm slice-thickness) DICOM images were annotated by a radiologist using semi-automated segmentation tools contained in the Vitrea software suite (Vital Images, Toshiba group), with separate binary mask annotations of the following tissues: muscle, body fat, aorta, vertebral column, epicardial fat, heart, and lungs.

### Human defined-feature image analysis methods

A total of 16,210 image features were defined to assess the segmented tissues and organs. These features were broadly divided into three groups: I) intensity based features, II) texture based features, and III) evidence based features. Intensity features relate to the first order statistics of the intensity histogram, quantifying the density of the tissue. The texture based features include the first and second order matrix statistics of the grey level co-occurrence (GLCM), grey level run length (GLRLM), grey level size zone (GLSZM), and multiple grey level size zone (MGLSZM) matrices. These features were computed over nine directions, at 45 degree intervals along each cardinal axis of the 3-D volume. The intensity and texture based features were extracted using the Radiomics package implemented in R (J. Carlson, https://cran.r-project.org/web/packages/radiomics/).

The evidence-based features were engineered to reflect the pre-existing knowledge of imaging biomarkers in the radiology literature. Cardiac and aortic calcification was quantified using pixel value bins, as per the Agatston methodology^31^. Due to the presence of IV contrast in the images, results were excluded when the average density of the heart or aorta segment was higher than the lower bound of the calcification density bin (for example, when the aortic average density was 250 Hounsfield units (HU), we excluded “calcification” pixel values below 300 HU). This resulted in many missing data points in the calcification values, particularly at lower density levels.

The degree of pulmonary emphysema was quantified by the presence and extent of low attenuating areas (LAA%)^32^. Bone mineral density^33^ was quantified by first order statistical analysis of the intensity (density) of medullary bone, after the exclusion of cortical bone from the vertebral column segment using a density threshold. The slice area and overall volume of each tissue was calculated, reflecting the literature that the dimensions of various tissues (e.g., heart size) are predictive of mortality^58^.

The task agnostic features were adapted from previous work^34^ where the methods were applied to localised regions of medical scans, such as in the analysis of small tumours. This task is quite different from the analysis of large organs and tissues, and we expected that the variation in these features over space would be useful in the prediction of disease (for example, the craniocaudal distribution of low attenuation areas in emphysema). To account for these spatial variations the average of each feature was calculated across the whole tissue, as well as within spatial quartiles along each axis. A spatial weighted mean was also calculated for each feature.

The division of features between tissues was as follows: 2506 (aorta), 2506 (heart), 2236 (lungs), 2182 (epicardial fat), 2182 (body fat), 2182 (muscle), 2416 (bone), where 1310 of the total 16,210 features represent evidence-based features. A total of 253 features were excluded for containing missing values, all of which were from the lower density bins of the cardiac and aortic calcification feature set. There were 15,957 remaining image features.

### Statistical analysis and predictive models for human-defined features

In order to analyse the large number of features extracted, standard statistical techniques used in high-dimensional data analysis (e.g., genomics) were applied. A logistic regression model was fitted to each feature separately, conditioned to account for the pair matched study design. The clinical relevance of each covariate was measured by the p-value, with a significance threshold (α) of <0.05 (i.e., −log(p) >3). The significance and distribution of image features were visualised with Manhattan plots.

A variety of feature selection methods were tested to inform a simplified risk score. These included principal component analysis (PCA)^59^, L1-constrained regularisation (the Lasso)^60^, selection methods based on univariate model significance, and minimum redundancy-maximum relevance (mRMR)^50^. The use of mRMR with 5 selected features was felt to best balance coverage of features from multiple tissues with the risk of overfitting our data. These continuous features were standardised with z-scoring, then summed to form our simplified risk score. This phenotypic score was dichotomised at the median to create “high risk” and “low risk” phenotypic groups. We present raw mortality rates for each phenotype and Kaplan-Meier curves to demonstrate the discriminative ability of the score.

The exploration of the feature distributions and the mortality risk score survival models were both performed on the entire dataset (training and testing data), as neither process involved model selection or hyperparameter tuning.

Mortality classification was performed to assess the predictive capacity of the engineered features using a variety of standard classifiers including linear and non-linear support vector machines, random forests, and various boosted tree methods. Model selection was performed via cross-validation within the training data.

Due to computational constraints, only the random forest classifier was applied to the full set of 15,957 engineered image features. For the other classification methods, two feature selection approaches were implemented with Lasso regression and principal components analysis. Selection with the Lasso within each fold identified between 4 and 20 features. PCA identified 16 principal components accounting for 95% of the variation in the data.

The most performant model was the random forest classifier without feature selection (i.e., with all of the human-crafted features as the input), with 150 trees and 16 variables randomly sampled at each split.

### Deep learning methodology

A convolutional neural network (ConvNet) was designed to predict all-cause mortality. While the architecture of this network was informed by current standard practices in the field, several alterations were required to deal with the unique aspects of CT image data.

**Figure 5:**
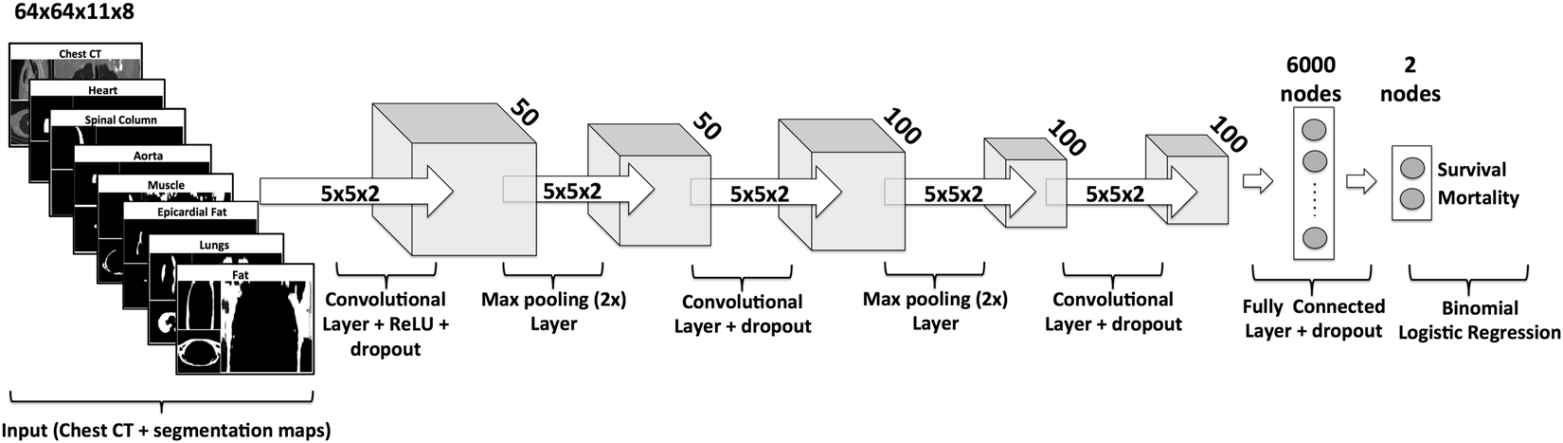
Schematic of the deep learning architecture for the mortality classification task.

The basic ConvNet architecture was determined by model selection using the training data. We explored a range of combinations of layer depth, layer size, and various combinations of non-linearities. The selected model (see Fig. 5) used in this work had three convolutional layers with 50 filters in the first layer and 100 filters in the second to fourth layers, where the filters had size 5 × 5 × 2. There were max pooling operations after the first and second convolutional layers, with a pooling size of 2 × 2 × 2. The first convolutional layer had rectified linear units (ReLU)^61^. After the third convolutional layer there was a fully connected layer containing 6,000 nodes, and the output layer had two nodes with softmax activation. For training, dropout of 0.35 was applied in all layers^62^. The learning rate started at 0.0005, from epochs 1 to 10, which was then continuously reduced until it reached 0.00001 from epoch 60 to 120. We optimised with RMS prop^63^ with *ρ* = 0.9.

We determined the optimal architecture for this deep learning model empirically, testing numerous variants. In particular we note that changing the depth of the network reduced performance, and the size of the fully connected layer was positively associated with prediction accuracy. We did not increase our model depth beyond 6,000 hidden units due to computational constraints.

Cross-sectional medical image volumes are much larger than the images that are usually analysed with ConvNets. To reduce the complexity of the problem, the large CT volumes (512 × 512 pixels, with 50–70 slices per case depending on the length of the patients’ lungs) were downsampled to 64 × 64 × 11 volumes using bicubic interpolation. 3d convolutions were implemented to manage the volumetric nature of the data, in keeping with the general practice in computer vision research of matching the dimensions of the convolutions to the data. The addition of the seven binary segmentation masks as channel inputs in the model was intended to promote the learning of anatomy based models for the prediction task: the distribution of tissues which contain predictive but very different features. For instance, the features that predict mortality in the lungs are assumed to be different from the predictive features in the vertebral bodies. With the segmentation maps the dimensions of the input tensor for each case were 64 × 64 × 11 × 8, where the final dimension is not spatial but is incorporated as a “channel” for each pixel (similar to RGB channels in colour photographs).

**Figure 6:**
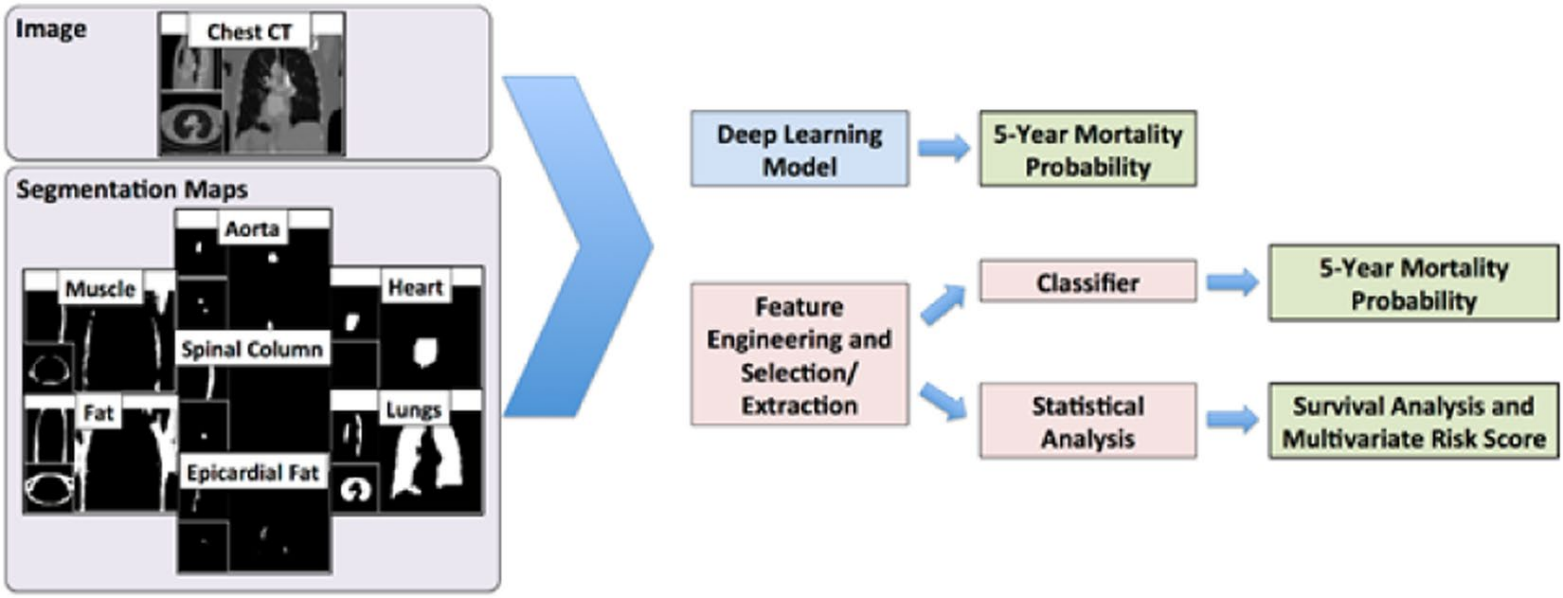
Diagrammatic representation of the analysis pipelines with engineered features and deep learning methods.

The feature engineering and deep learning analysis pipelines are demonstrated in Fig. 6.

### Classification experiments

We assess the predictive performance of the feature engineering and deep learning methodologies based on a 6-fold cross-validation experiment, where we form six training sets, each containing 40 cases, and six testing sets, each with eight cases with no overlapping between training and testing sets in each fold. The classification performance is measured using the receiver operating characteristic (ROC) curve and area under the ROC curve (AUC)^64^ using the classifier confidence on the 5-year mortality classification, as well as the mean accuracy across the 6 experiments.

### Visual analysis

Qualitative visual comparison was made between the cases correctly identified as mortality or survival cases by the deep learning and feature engineering models. The strongest predictions averaging the results of the two models were identified and reviewed by a consultant radiologist. This was performed to demonstrate “face validity”, that the model predictions can identify similar patterns and patients as the assessment of a clinical expert.

### Data availability

The datasets generated during and/or analysed during the current study are not publicly available due to the clinical and confidential nature of the material but can be made available from the corresponding author on reasonable request.

## Electronic supplementary material

Supplementary Figures

## References

[1] Collins FS, Varmus H (2015). A new initiative on precision medicine. New England Journal of Medicine.

[2] Wild CP (2005). Complementing the genome with an “exposome”: the outstanding challenge of environmental exposure measurement in molecular epidemiology. Cancer Epidemiology Biomarkers & Prevention.

[3] Colburn W (2001). Biomarkers and surrogate endpoints: Preferred definitions and conceptual framework. Biomarkers Definitions Working Group. Clinical Pharmacol & Therapeutics.

[4] Wallace, K. *et al*. Report on the NCSS Fact Finding Cardiotoxicity Expert Working Group. http://www.fda.gov/ohrms/dockets/ac/01/briefing/3798b1_04_holt/sld005.htm (2001).

[5] Dancey JE, Bedard PL, Onetto N, Hudson TJ (2012). The genetic basis for cancer treatment decisions. Cell.

[6] McCarthy JJ, McLeod HL, Ginsburg GS (2013). Genomic medicine: a decade of successes, challenges, and opportunities. Science translational medicine.

[7] AIHW, E. (Australia’s Health Series, Australian Institute of Health and Welfare (AIHW), Canberra, Australia, 2014).

[8] Organization, W. H. Preventing chronic diseases: a vital investment: WHO global report. (2005).

[9] Rappaport SM, Smith MT (2010). Environment and disease risks. Science.

[10] Rappaport SM (2011). Implications of the exposome for exposure science. Journal of Exposure Science and Environmental Epidemiology.

[11] Wilkens, L. R. & Lee, J. *Nutritional epidemiology*. (Wiley Online Library, 1998).

[12] Dishman, R., Heath, G. & Lee, I.-M. *Physical activity epidemiology*. 39–49 (Human Kinetics, 2004).

[13] Applegate WB, Blass JP, Williams TF (1990). Instruments for the functional assessment of older patients. New England Journal of Medicine.

[14] Hripcsak G, Albers DJ (2013). Next-generation phenotyping of electronic health records. Journal of the American Medical Informatics Association.

[15] Gligorijević V, Malod‐Dognin N, Pržulj N (2016). Integrative methods for analyzing big data in precision medicine. Proteomics.

[16] Sonnenburg JL, Bäckhed F (2016). Diet-microbiota interactions as moderators of human metabolism. Nature.

[17] Aronson SJ, Rehm HL (2015). Building the foundation for genomics in precision medicine. Nature.

[18] Prasad V, Fojo T, Brada M (2016). Precision oncology: origins, optimism, and potential. The Lancet Oncology.

[19] Fox JL (2015). Obama catapults patient-empowered Precision Medicine. Nature biotechnology.

[20] Butte, A. J. It takes a genome to understand a village: Population scale precision medicine. *Proceedings of the National Academy of Sciences*, 201615329 (2016). 10.1073/pnas.1615329113 PMC5098617 27791179

[21] Civelek M, Lusis AJ (2014). Systems genetics approaches to understand complex traits. Nature Reviews Genetics.

[22] Wei W-Q, Denny JC (2015). Extracting research-quality phenotypes from electronic health records to support precision medicine. Genome medicine.

[23] Toll D, Janssen K, Vergouwe Y, Moons K (2008). Validation, updating and impact of clinical prediction rules: a review. Journal of clinical epidemiology.

[24] Yourman LC, Lee SJ, Schonberg MA, Widera EW, Smith AK (2012). Prognostic indices for older adults: a systematic review. Jama.

[25] Adlassnig K-P (1986). Fuzzy set theory in medical diagnosis. IEEE Transactions on Systems, Man, and Cybernetics.

[26] Boyd, S. & Vandenberghe, L. *Convex optimization*. (Cambridge university press, 2004).

[27] Collinson P, Troponin t (1998). T or troponin I or CK-MB (or none?). European heart journal.

[28] Drucker E, Krapfenbauer K (2013). Pitfalls and limitations in translation from biomarker discovery to clinical utility in predictive and personalised medicine. EPMA journal.

[29] Poste G (2011). Bring on the biomarkers. Nature.

[30] Bush WS, Oetjens MT, Crawford DC (2016). Unravelling the human genome-phenome relationship using phenome-wide association studies. Nature Reviews Genetics.

[31] Greenland P, LaBree L, Azen SP, Doherty TM, Detrano RC (2004). Coronary artery calcium score combined with Framingham score for risk prediction in asymptomatic individuals. Jama.

[32] Haruna A (2010). CT scan findings of emphysema predict mortality in COPD. CHEST Journal.

[33] Johnell O (2005). Predictive value of BMD for hip and other fractures. Journal of bone and mineral research.

[34] Aerts, H. J. *et al*. Decoding tumour phenotype by noninvasive imaging using a quantitative radiomics approach. *Nature communications* **5** (2014). 10.1038/ncomms5006 PMC4059926 24892406

[35] Kato H (2007). Computer-aided diagnosis of hepatic fibrosis: preliminary evaluation of MRI texture analysis using the finite difference method and an artificial neural network. American Journal of Roentgenology.

[36] Ito M (1995). Trabecular texture analysis of CT images in the relationship with spinal fracture. Radiology.

[37] Gillies RJ, Kinahan PE, Hricak H (2015). Radiomics: images are more than pictures, they are data. Radiology.

[38] Kumar V (2012). Radiomics: the process and the challenges. Magnetic resonance imaging.

[39] Forsyth, D. A. & Ponce, J. *Computer vision: a modern approach*. (Prentice Hall Professional Technical Reference, 2002).

[40] Palmer LJ, Cardon LR (2005). Shaking the tree: mapping complex disease genes with linkage disequilibrium. The Lancet.

[41] Fu W, D O’Connor T, Akey JM (2013). Genetic architecture of quantitative traits and complex diseases. Current opinion in genetics & development.

[42] LeCun Y, Bengio Y, Hinton G (2015). Deep learning. Nature.

[43] Zeiler, M. D. & Fergus, R. In *European Conference on Computer Vision*. 818–833 (Springer).

[44] Gulshan V (2016). Development and validation of a deep learning algorithm for detection of diabetic retinopathy in retinal fundus photographs. JAMA.

[45] Esteva A (2017). Dermatologist-level classification of skin cancer with deep neural networks. Nature.

[46] Charlson ME, Pompei P, Ales KL, MacKenzie CR (1987). A new method of classifying prognostic comorbidity in longitudinal studies: development and validation. Journal of chronic diseases.

[47] Brady Z, Cain TM, Johnston PN (2011). Paediatric CT imaging trends in Australia. Journal of medical imaging and radiation oncology.

[48] de González AB (2009). Projected cancer risks from computed tomographic scans performed in the United States in 2007. Archives of internal medicine.

[49] Ganna A, Ingelsson E (2015). 5 year mortality predictors in 498 103 UK Biobank participants: a prospective population-based study. The Lancet.

[50] Peng H, Long F, Ding C (2005). Feature selection based on mutual information criteria of max-dependency, max-relevance, and min-redundancy. IEEE Transactions on pattern analysis and machine intelligence.

[51] Breiman L (2001). Random forests. Machine learning.

[52] D’Agostino RB, Grundy S, Sullivan LM, Wilson P (2001). Validation of the Framingham coronary heart disease prediction scores: results of a multiple ethnic groups investigation. Jama.

[53] Rietbrock S, Heeley E, Plumb J, van Staa T (2008). Chronic atrial fibrillation: Incidence, prevalence, and prediction of stroke using the Congestive heart failure, Hypertension, Age >75, Diabetes mellitus, and prior Stroke or transient ischemic attack (CHADS2) risk stratification scheme. American heart journal.

[54] Antman EM (2000). The TIMI risk score for unstable angina/non–ST elevation MI: a method for prognostication and therapeutic decision making. Jama.

[55] Hanley JA, McNeil BJ (1982). The meaning and use of the area under a receiver operating characteristic (ROC) curve. Radiology.

[56] Sham PC, Purcell SM (2014). Statistical power and significance testing in large-scale genetic studies. Nature Reviews Genetics.

[57] Greenspan H, van Ginneken B, Summers RM (2016). Guest Editorial Deep Learning in Medical Imaging: Overview and Future Promise of an Exciting New Technique. IEEE Transactions on Medical Imaging.

[58] Pocock SJ (2006). Predictors of mortality and morbidity in patients with chronic heart failure. European heart journal.

[59] Hotelling H (1933). Analysis of a complex of statistical variables into principal components. Journal of educational psychology.

[60] Tibshirani, R. Regression shrinkage and selection via the lasso. *Journal of the Royal Statistical Society*. *Series B* (*Methodological*), 267–288 (1996).

[61] Nair, V. & Hinton, G. E. In *Proceedings of the 27th International Conference on Machine Learning* (*ICML-10*). 807–814.

[62] Srivastava N, Hinton GE, Krizhevsky A, Sutskever I, Salakhutdinov R (2014). Dropout: a simple way to prevent neural networks from overfitting. Journal of Machine Learning Research.

[63] Dauphin, Y., de Vries, H., Chung, J. & Bengio, Y. RMSProp and equilibrated adaptive learning rates for non-convex optimization (2015). arXiv preprint. *arXiv preprint arXiv*:*1502*.*04390*.

[64] Bradley AP (1997). The use of the area under the ROC curve in the evaluation of machine learning algorithms. Pattern recognition.

