# Supplementary material for: Precision Radiology: Predicting longevity using feature engineering and deep learning methods in a radiomics framework
Source: Sci Rep. 2017 May 10;7:1648. doi: 10.1038/s41598-017-01931-w (PMC5431941; doi:10.1038/s41598-017-01931-w)
Supplement: Supplementary file 1 — Supplementary Figures [file 41598_2017_1931_MOESM1_ESM.pdf]

Supplemental Figures

Precision Radiology: Predicting longevity using feature engineering and deep learning methods in a radiomics framework

Dr Luke Oakden-Rayner[1,2] (FRANZCR)*, Associate Professor Gustavo Carneiro[3] (PhD), Associate Professor Taryn Bessen[1] (FRANZCR PhD), Assistant Professor Jacinto C. Nascimento[4] (PhD), Professor Andrew P. Bradley[5] (PhD), Professor Lyle J. Palmer[2] (PhD FRSS)

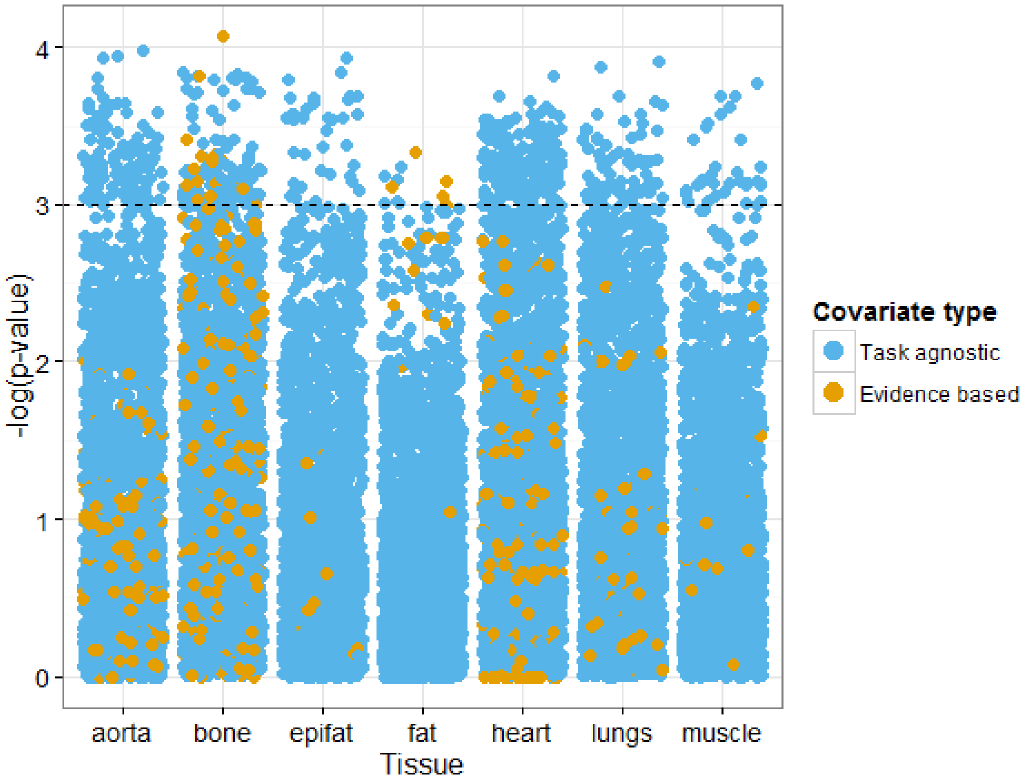

Supplemental figure 1: Distribution of "evidence-based" imaging features.
Manhattan plot showing the number of covariates in each tissue segment, highlighting the distribution of "evidence-based" covariates. The dotted line identifies the threshold of significance (the -$\log_{10}$ of the p-value is plotted on the y-axis, covariates above this line have p-values < 0.05).

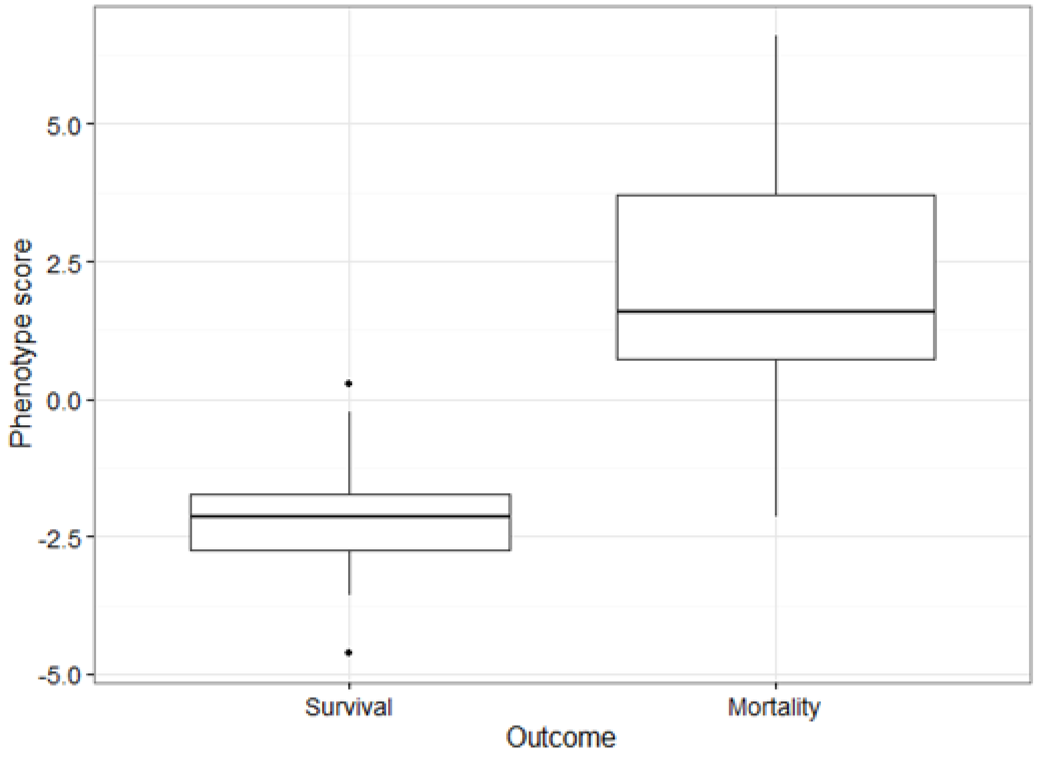

Supplemental figure 2: Distribution of phenotypic scores.
Box and whisker plot showing the distribution of raw (not dichotomised) mortality phenotype scores for the mortality cases and controls.

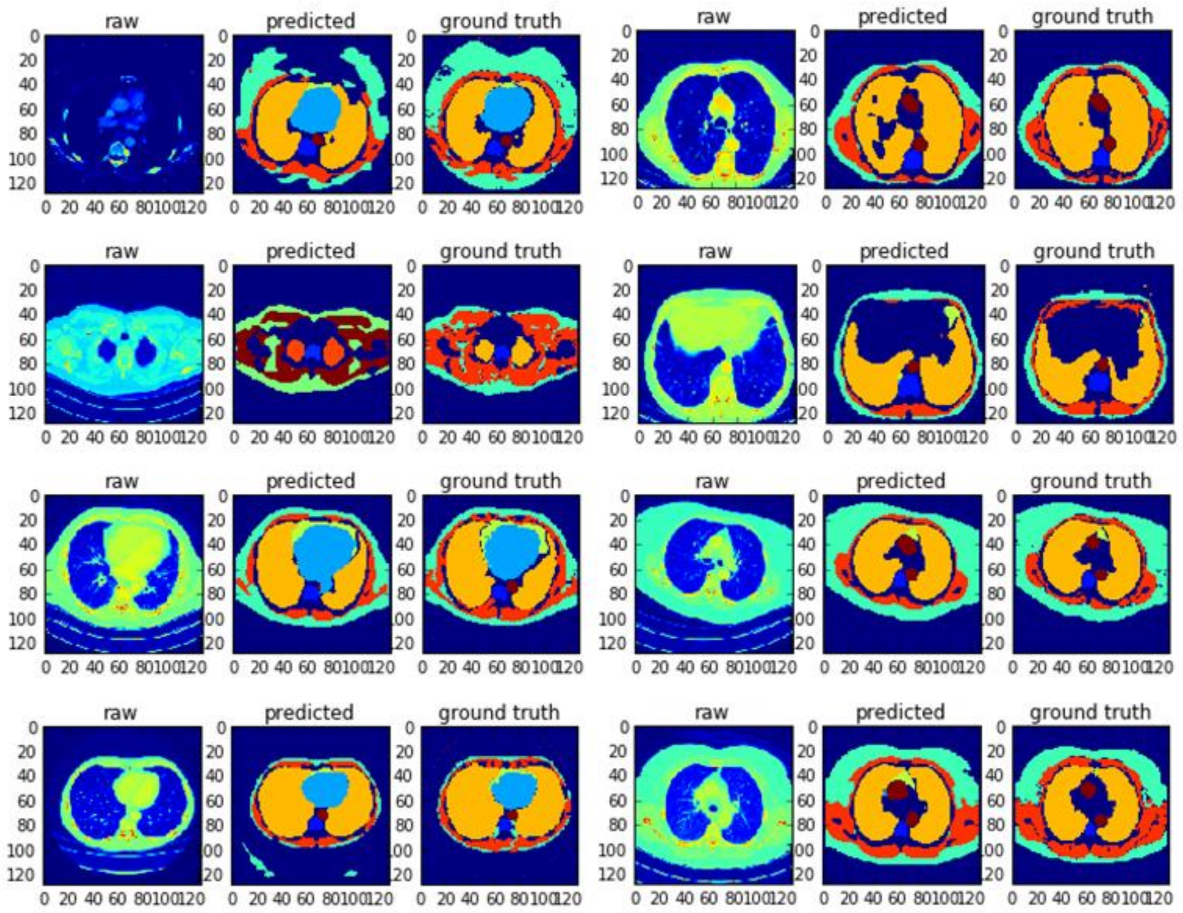

Supplemental Figure 3: Visual demonstration of early work on an automated segmentation system.

Preliminary results using deep learning to perform automatic tissue segmentation. Randomly selected CT slices (labelled 'raw') are drawn from a held-out test set of 8 cases, the segmentation system was trained on the remaining 40 cases. The automated segmentations (labelled 'predicted') and the original expert hand segmentations (labelled 'ground truth') are presented side by side for visual comparison.
